# Development and validation of prediction model to estimate 10-year risk of all-cause mortality using modern statistical learning methods: a large population-based cohort study and external validation

**DOI:** 10.1186/s12874-020-01204-7

**Published:** 2021-01-06

**Authors:** Olesya Ajnakina, Deborah Agbedjro, Ryan McCammon, Jessica Faul, Robin M. Murray, Daniel Stahl, Andrew Steptoe

**Affiliations:** 1 Department of Behavioural Science and Health, Institute of Epidemiology and Health Care, University College London, London, UK; 2 Department of Biostatistics & Health Informatics, Institute of Psychiatry, Psychology and Neuroscience, King’s College London, London, UK; 3 Survey Research Center, Institute for Social Research, University of Michigan, Ann Arbor, USA; 4 Department of Psychosis Studies, Institute of Psychiatry, Psychology and Neuroscience, King’s College London, London, UK; 5 Department of Psychiatry, Experimental Biomedicine and Clinical Neuroscience (BIONEC), University of Palermo, Palermo, Italy

**Keywords:** Mortality, Survival, Prognostic factors, Statistical learning, Absolute risk, Population-based longitudinal study

## Abstract

**Background:**

In increasingly ageing populations, there is an emergent need to develop a robust prediction model for estimating an individual absolute risk for all-cause mortality, so that relevant assessments and interventions can be targeted appropriately. The objective of the study was to derive, evaluate and validate (internally and externally) a risk prediction model allowing rapid estimations of an absolute risk of all-cause mortality in the following 10 years.

**Methods:**

For the model development, data came from English Longitudinal Study of Ageing study, which comprised 9154 population-representative individuals aged 50–75 years, 1240 (13.5%) of whom died during the 10-year follow-up. Internal validation was carried out using Harrell’s optimism-correction procedure; external validation was carried out using Health and Retirement Study (HRS), which is a nationally representative longitudinal survey of adults aged ≥50 years residing in the United States. Cox proportional hazards model with regularisation by the least absolute shrinkage and selection operator, where optimisation parameters were chosen based on repeated cross-validation, was employed for variable selection and model fitting. Measures of calibration, discrimination, sensitivity and specificity were determined in the development and validation cohorts.

**Results:**

The model selected 13 prognostic factors of all-cause mortality encompassing information on demographic characteristics, health comorbidity, lifestyle and cognitive functioning. The internally validated model had good discriminatory ability (*c*-index=0.74), specificity (72.5%) and sensitivity (73.0%). Following external validation, the model’s prediction accuracy remained within a clinically acceptable range (*c*-index=0.69, calibration slope *β*=0.80, specificity=71.5% and sensitivity=70.6%). The main limitation of our model is twofold: 1) it may not be applicable to nursing home and other institutional populations, and 2) it was developed and validated in the cohorts with predominately white ethnicity.

**Conclusions:**

A new prediction model that quantifies absolute risk of all-cause mortality in the following 10-years in the general population has been developed and externally validated. It has good prediction accuracy and is based on variables that are available in a variety of care and research settings. This model can facilitate identification of high risk for all-cause mortality older adults for further assessment or interventions.

**Supplementary Information:**

The online version contains supplementary material available at 10.1186/s12874-020-01204-7.

## Background

Rapid population ageing is a worldwide phenomenon, highlighting an emergent need to have a reliable prediction model for estimating an individual mortality risk. Similar to the prediction tools for coronary heart disease [1], breast cancer [2] and cardiovascular disease [3], which are now included in clinical guidelines for therapeutic management, a prediction model for all-cause mortality in older people can be used to communicate risk to individuals and their families (if appropriate) and guide strategies for risk reduction.

Recently, closely following the methods developed by American counterparts [4–6], a 10-item index was derived for predicting a 10-year mortality risk in adults aged 50–101 years old living in England [7]. Drawing on stepwise regression for model building and utilising complete-cases, the 10-item index was reported to have an excellent ability to identify older individuals with low- and high-risk for all-cause mortality in the next 10 years [7]. However, because stepwise regressions are known to lead to overfitting and poor prediction of new cases [8], the index may not predict all-cause mortality equally well when applied to a new sample [9]. Considering the average life expectancy in England is 81 years old [10], it is possible that high discriminative ability of the 10-item index was a result of including adults who were likely to die in the next 10 years due to their old age. The authors also did not provide information about the model calibration precluding estimating probabilities of all-cause mortality, which are necessary for informed clinical decision making [11, 12].

In the era of precision medicine, more computationally demanding modern statistical learning algorithms, particularly regularised regression methods (RRMs), are suggested as optimal methods for clinical and personalised risk prediction [13], as they are able to overcome the weaknesses of stepwise regressions. Therefore, using the same dataset, as in the 10-item index of mortality [7], but restricting individuals to a more representative age, we employed RRMs to develop a new model to predict risk of all-cause mortality over a 10-year period. To ensure our model is appropriate for routine use in clinical practice [14], we externally validated it. Although direct comparison with the 10-item index of mortality will be difficult because of differences in the data handling and reporting, to aid understanding if our model offered an improved prediction efficiency, we externally validated the 10-item index [7], which has not been done before.

## Methods

### Data sources and study population

For this study, we used data from England to develop our mortality model and data from United States to externally validate it. To ensure that the cohorts employed were as representative of the general populations as possible, we did not limit them based on their help and health statuses.

#### Derivation cohort (England)

The data for model development came from the English Longitudinal Study of Ageing (ELSA), which is multidisciplinary study of a nationally representative sample of the English population aged ≥50 years [15]. The ELSA study started in 2002–2003 (wave 1) with *n*=11,156 participants recruited from the Health Survey for England (HSE), which was an annual cross-sectional survey designed to monitor the health of the general population. As the inclusion criteria were being a member of a participating household from HSE in which at least one person had agreed to follow-up, born before 1 March 1952 and living in a private household in England at the time of the first wave of fieldwork, the ELSA sample was restricted to participants living in the community [15]. Comparisons of ELSA with the national census showed that the baseline sample was representative of the non-institutionalised general population aged ≥50 in the UK [15]. Subsequently, this sample was followed-up every two years. In the present study, wave 1 formed our baseline and follow-up data were obtained from wave 6 (2012–2013). To limit the overriding influence of age in a “cohort of survivors”, we excluded participants who were > 75 years old.

#### Validation cohort (United States)

The external validation of our model was performed using data from the Health and Retirement Study (HRS) [16], which is a nationally representative, biannual longitudinal survey of adults ≥50 years old residing in the United States (US). Since ELSA was developed as a companion study to the HRS facilitating opportunities for cross-national analyses [15], the HRS sample was also restricted to participants living in the community. The HRS encompasses the detailed information collected on respondents’ characteristics and death recorded from an exit interview with a relative or proxy [17]. A more detailed description of the HRS sample is provided in Supplementary Materials. For the purpose of validating our mortality model, we included information on mortalities that occurred from 30 January 2004 to 1 August 2015 giving us a 10-year follow-up period, which is in line with the derivation cohort. To make the external sample more consistent with the derivation data, we further limited it to those who were aged 50–75 years old.

### Outcome

The outcome was all-cause mortality that occurred from 2002 to 2003 through to 2013, which was ascertained from the National Health Service central register, which captures all deaths occurring in the UK. All participants included in this study provided written consent for linkage to their official records. Survival time was defined as the period from baseline when all ELSA participants were alive to the date when an ELSA participant was reported to have died during the 10-year follow-up. For those who did not die during follow-up, the survival time was calculated using the period spanning from baseline until the end of the study.

### Prognostic factors

114 prognostic factors related to participants’ general health, comorbid health conditions, mental health, cognitive domains, life satisfactions, mobility, physical activity, social-economic status and social relationships were considered for the model development (Additional file 1). Following a previous research protocol [18], we excluded prognostic factors shown to have a high collinearity with other variables, or had > 50% missing values (Additional file 2); 84 prognostic factors were retained for the analyses.

### Power calculations

To ensure we had sufficient power to develop a prediction model for all-cause mortality accurately, we calculated a sample size required for development a prediction model according to the recent guidelines [19]. Accordingly, we estimated all-cause mortality events in the derivation cohort that occurred during the 10-year follow-up period; since all variables included in the Cox-Lasso regression were either continuous or binary, we included 84 degrees of freedom worth of prognostic factors (i.e., parameters) in the power calculations. Assuming the value of R^2^ corresponds to an R^2^_Nagelkerke_ of 0.15 (i.e., R^2^_CS_ =0.15* × max((R^2^_CS_)) [19] sample size required for our new model development was *n*=8978 with 1118 outcome events. These power calculations highlighted that we had an effective sample size of 15 mortality events per predictor, which is higher than with the recommended 13.3 cases per each predictor as estimated using calculations developed by Riley et al. [19]. We have presented the sample size calculations in Additional file 3.

### Statistical analysis

The process of model development, evaluation and validation was carried out according to methodological standards outlined by Steyerberg et al. [13]; results were reported according to the Transparent Reporting of a multivariable prediction model for Individual Prognosis Or Diagnosis (TRIPOD) guidelines [20]. A more detailed description of these methods is provided in the Supplementary Methods.

#### Imputation of missing values

The missing data were imputed using missForest, which is a nonparametric imputation method based on random forest [21]; it handles continuous and categorical variables equally well and accommodates non-linear relation structures [21, 22]. Distribution of the variables before and after imputation is presented in Additional file 3.

#### Variable selection and model fitting

To build the prediction model and identify which of 84 prognostic factors were important for estimating an individual risk of all-cause mortality during the 10-year follow-up, we applied Cox proportional hazards model with regularisation by the least absolute shrinkage and selection operator (Cox-Lasso) [23]. Cox-Lasso entails fitting a model, which, by imposing penalty (*λ*) on the size of regression parameter estimates to shrink them towards 0 [24, 25], simultaneously selects predictors, estimates their effects and introduces parsimony. Therefore, if a suitable *λ* is chosen, Cox-Lasso automatically performs variable selection and deals with collinearity. Selection of the tuning parameter *λ* optimising the model performance is described below.

#### Model estimation

The tuning parameter *λ* optimising the partial log-likelihood was chosen from a grid of 100 *λ* values through 10-fold repeated cross-validation (CV) [23]. 10-fold CV divided data randomly into 10 non-overlapping data partitions; individuals included in the first 9 partitions were considered as the test sample, and the remaining individuals as the training sample. To reduce a potential variance, 10-fold CV was repeated 100 times computing the partial log-likelihood for each *λ* value. The optimal *λ* was chosen as the one that generated the largest partial log-likelihood. The model that corresponded to the optimal *λ* was referred to as “model_best_”. As model_best_ may still select a large proportion of noise variables, even if their inclusion leads to an optimal performance [25], *λ* that had a partial log-likelihood within one-standard error (SE) of the maximum was suggested to be a better compromise between a higher proportion of true prognostic factors among the selected predictors and good prediction accuracy [26]. We referred to this model as “model_1-SE_”. As a parsimonious model is desirable for practice [27] and may generalise better to different populations [28], though often at the expense of a lower predictive performance, we additionally considered a model with a stronger penalty that had a partial log-likelihood within 3% of the optimum partial-log likelihood (“model_3%_”), yielding more parsimony [24].

#### Model performance

Models’ accuracy was measured with discrimination and calibration. Discrimination indicates how well a model separates individuals who experienced an event from those who did not; we assessed discrimination using concordance index (*c*-index) [29]. Calibration, assessed with the calibration slope *β* (which is one if the predicted risks are not too extreme or too moderate), describes how well the predicted survival corresponds to the survival from the observed data [11, 12] and can be described as a measure of bias in a model [29]. We further measured the prediction accuracy of our models at 10 years with sensitivity and specificity. Unlike the traditional 50%, which follows often incorrect assumption that the false-positive and false-negative are equally important [13], to classify an individual as high or low risk based on a prediction model, a cutoff for the predicted probability (i.e., “decision threshold”) [13] was selected by maximizing the sum of the model’s sensitivity and specificity to minimize the false positives, which are unavoidable [30]. This entailed selecting the decision threshold that maximized the overall correct classification rates, while choosing the point on the receiver operating characteristic (ROC) curve farthest from chance [31]. The results for the models’ performances for the traditional 50% and the best threshold are provided in Additional files 6, 9 and 10.

#### Model internal validity

The models’ performances before internal validation are presented in Additional file 7. To correct measures of predictive performance for optimism (difference in test performance and apparent performance) [32], which occurs when a model’s predictions are more extreme than they should be for individuals in a new dataset from the same target population, we carried out internal validation of our model using Harrell’s optimism-correction procedure [29] in the derivation cohort. Accordingly, the whole model building process from imputing the missing values with missForest, selecting tuning parameter *λ* through repeated CV to fitting Cox-Lasso is repeated 1000 times on different resamples. However, due to high computational demands of missForest algorithm when applied to our large sample, we performed imputation first, as suggested [33], followed by full validation of Cox-Lasso through Harrell’s optimism correction procedure as outlined above. We then estimated the overall optimism across all models (Additional file 6), which was minimal negating a need for a recalibration. To account for over-fitting during the development process, for each measure of performance (*p*), we obtained the optimism-corrected performance (*p*_corrected_), by using the formula: *p*_corrected_=*p*_apparent_-*p*_optimism_ [13] (Additional files 8 and 10).

#### Model external validity

**Fig. 1:**
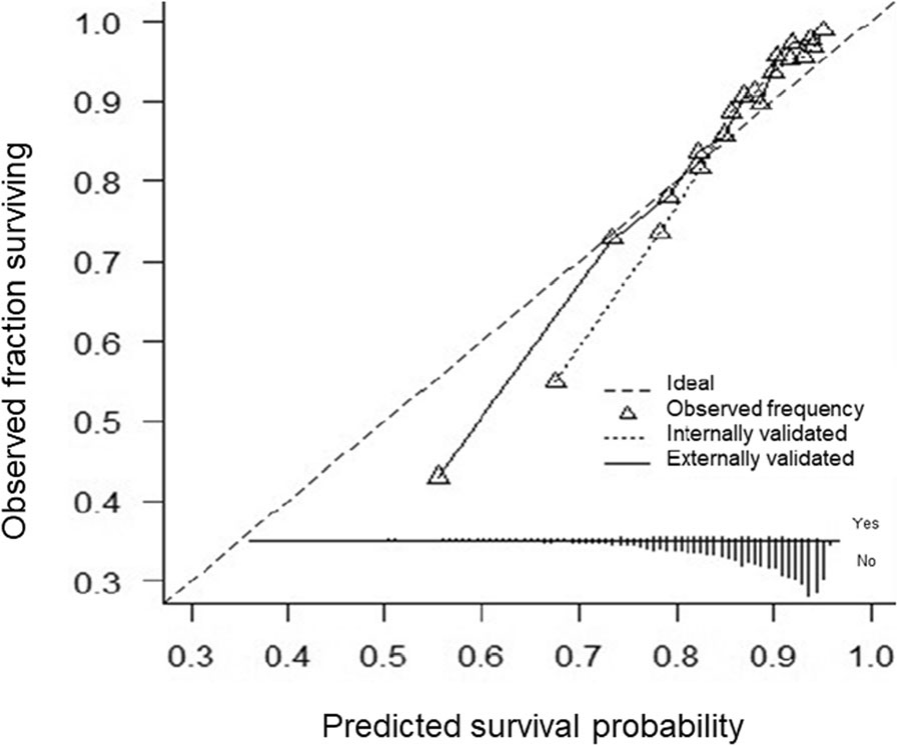
Calibration plot presenting agreement between the predicted and observed survival rates at 10-years as estimated by our newly developed model

We applied our risk prediction model to each participant in the external validation cohort (Additional files 11 and 12). Distributions of the variables included in the final all-cause mortality model in derivation cohort (ELSA) and validation cohort (HRS) are presented in Additional file 13. The proportion of missingness in these variables varied from 0 to 3.7%. Therefore, before externally validated our model using the data from the HRS study, we imputed the missing values in the validation cohort with missForest following the procedure as outlined for the derivation cohort. We examined the performance of our final model in the validation cohort by calculating the *c*-statistic, calibration slope, sensitivity and specificity as described above. A calibration plot was computed to assess graphically the agreement between the 10-year survival probability as predicted by the internally and externally validated models [13]; it is presented in Fig. 1. We estimated the risk equation for predicting the absolute hazard of all-cause mortality at 10 years, which is the exponential of the sum of an individual’s prognostic factors weighted by *β*-estimates from Cox-Lasso [34], multiplied by the baseline hazard estimate at 10 years. We could therefore derive and present an equation for the predicted absolute risk of all-cause mortality during a 10-year period using the baseline survival, which is presented in Additional file 14. Further, as the main product of a Cox model is a prognostic index (PI), which represents a summary of the combined effects of an individual’s risk factors [34], we estimated PIs from the externally validated model and translated these into probabilities of all-cause mortality during the 10-year follow-up period (Table 4). The PIs were calculated using the linear predictor from the Cox-Lasso model weighted by the regression coefficients from our final model; higher PI values indicate a worse prognosis. To assist a comparison with our model, we externally validated 10-item index [7], using the HRS sample and evaluated it with discrimination, calibration, sensitivity and specificity. Here, as the original model was developed using complete cases only, we externally validated 10-item index without employing imputation of missing values. An all-cause survival model had not been published using data from the external validation; thus, this set of analyses is unique to the present study.

#### Model presentation

**Fig. 2:**
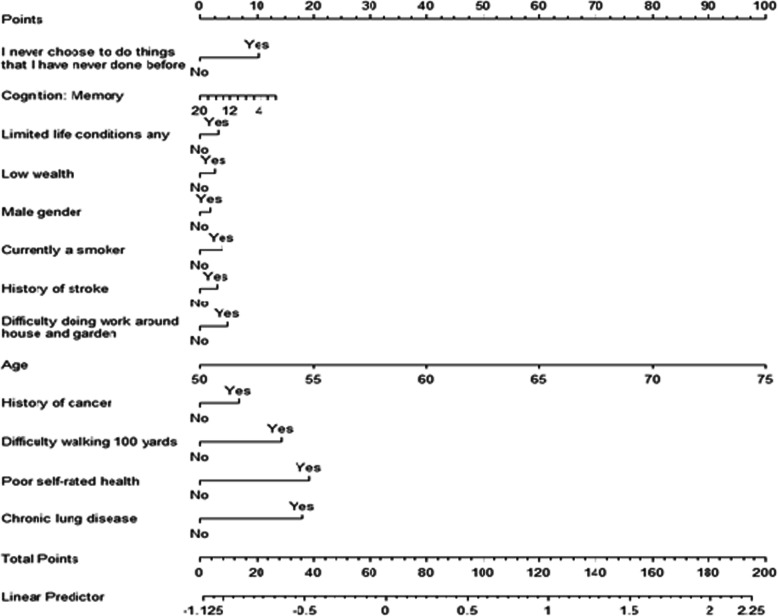
Nomogram for Cox-Lasso regression which enables calculating individual normalized prognostic indexes (PI, given by the linear predictor line) for all-cause mortality in the following 10 years. Coefficients are based on the Lasso-Cox model as estimated by the final model for the all-cause mortality. The nomogram allows computing the normalized prognostic index (PI) for a new individual. The PI is a single-number summary of the combined effects of a patient’s risk factors and is a common method of describing the risk for an individual. In other words, the PI is a linear combination of the risk factors, with the estimated regression coefficients as weights. The exponentiated PI gives the relative risk of each participant in comparison with a baseline participant (in this context the baseline participant would have value 0 for all the continuous covariates and being at the reference category for the categorical ones). The PI is normalized by subtracting the mean PI

The final model was presented as a nomogram [35] (Fig. 2), which allows an approximate graphical computation of a mathematical function to estimate individualised probability for all-cause mortality based on an individual’s characteristics. Nomogram has been shown to be better than clinician judgment in estimating an individual risk for an outcome [36].

## Results

### Study participants

For the model development, the sample comprised 9154 individuals; of these, 1240 (13.5%) died during the 10-year follow-up with an average length of survival of 70.2 months (SD_derivation_=35.4, range_derivation_=1–130). The baseline mean_derivation_ age for the entire sample was 61.5 years (SD_derivation_=7.2, range_derivation_=50–75); 46.6% were men and 96.7% were of white ethnicity. The sample for external validation comprised 2575 individuals; of these, 491 (19.1%) died during the 10-year follow-up with an average length of survival of 77.7 months (SD_validation_=36.5, range_validation_=1–135.9). The baseline mean_validation_ age for the entire sample was 62.7 years (SD_validation_=7.2, range_validation_=50–75); 43.1% were men and 81.0% were of white ethnicity.

### Model development and performance measures

Model_best_ selected 55 (65.5% of *n*=84), model_1-SE_ selected 54 (64.3% of n=84) and model_3%_ selected 13 (15.5% of n=84) prognostic factors (Table 1 and Additional file 5). To classify individuals at the high risk for all-cause mortality based on the selected variables, for model_best_ and model_1-SE_ the best decision threshold was estimated at 13.2%. The apparent performance statistics of all prediction models is presented in Additional file 6. Model_best_ and model_1-SE_ showed good internally validated discrimination (*c*-index_corrected_=0.75 for both models) and nearly perfect internally validated calibration (calibration slopes *β*_corrected_=1.06 and 1.07, respectively). For model_3%_ the best decision threshold was at 14.9%. After adjustment for optimism, model_3%_ was able to discriminate adults who died and did not die during the 10-year follow-up with a c-statistic of 0.74, and good specificity_corrected_ (72.5%) and sensitivity_corrected_ (73.0%); though, calibration slope *β*_corrected_ for model_3%_ was 1.64 (Table 2). Because the prediction model needs to be parsimonious to be practically manageable and calibration of 1.64 was within a range of previously reported models [37], we chose model_3%_ as our final model.

**Table 1:** Beta coefficients in prediction model developed using modern statistical learning methods to predict all-cause mortality in older people

Selected variables		Coefficients (log hazard ratios)	
***x***_**1**_	Age	***b***_**1**_	0.069544
***x***_**2**_	Never choose to do things I have never done before (1= “yes”)	***b***_**2**_	0.178672
***x***_**3**_	Cognition: Memory	***b***_**3**_	−0.011597
***x***_**4**_	Limited life conditions any (1= “yes”)	***b***_**4**_	0.053174
***x***_**5**_	Low wealth (1= “yes”)	***b***_**5**_	0.043483
***x***_**6**_	Male gender (1= “yes”)	***b***_**6**_	0.031585
***x***_**7**_	Currently a smoker (1= “yes”)	***b***_**7**_	0.068574
***x***_**8**_	History of stroke (1= “yes”)	***b***_**8**_	0.050877
***x***_**9**_	Difficulty doing work around house and garden (1= “yes”)	***b***_**9**_	0.086048
***x***_**10**_	History of cancer (1= “yes”)	***b***_**10**_	0.119762
***x***_**11**_	Difficulty walking 100 yards	***b***_**11**_	0.249760
***x***_**12**_	Poor self-rated health	***b***_**12**_	0.336850
***x***_**13**_	Chronic lung disease	***b***_**13**_	0.315754

**Table 2:** Prediction accuracy of the prediction model of 10-year risk for all-cause mortality developed using modern statistical learning methods

Models’ performances	Internally validated	Externally validated
*c*-index	0.74	0.69
Calibration slope	1.64	0.80
Sensitivity	72.5%	71.5%
Specificity	73.0%	70.6%

*SD* standard deviation

### Predictor variables and estimating an individual 10-year risk of mortality

Each additional year of age was associated with an increased individual 10-year mortality risk of 7% in relative terms (Table 1). Poor self-rated health, history of chronic lung disease and difficulty doing work around house and garden were the largest contributors to the risk for all-cause mortality, followed by an item concerning reluctance to do new things (an aspect of self-realisation), previous diagnoses of cancer, decreasing memory score, difficulty walking 100 yards and smoking. Risk for all-cause mortality further increased with low accumulated wealth, male gender, history of stroke and presence of a limiting longstanding illness. A worked example of calculating an absolute individualised risk for mortality in the following 10 years is provided in Table 3.

**Table 3:** Example calculation of an individual 10-year risk of all-cause mortality

*Patient description:*	
An individual is a male aged 75-years old, smoker who comes from a middle-class social-economic status. He has at least 1 limiting illness and previously had a cancer or a malignant tumour (excluding minor skin cancers) but has an intact memory. He stated that he never had a chance to do things that he never experienced before. The patient reports difficulties doing work around house and garden and struggles to walk 100 yards. Overall, he describes his health as poor; though he never experienced stroke and chronic lung disease.	
*Estimated Beta coefficient × variable for this person:*	
Using the nomogram (Fig. 1) and information from Table 2, we can estimate this patient’s probability to die in the following 10 years by adding points assigned in the nomogram to each factor in the model. Thus, in this example, the patient would have a total point score of 2 point (male gender) + 100 points (aged 75 years old) + 4 points (being a smoker) + 0 points (middle-class social-economic status) + 3 points (having at least one limiting illness) + 7 points (having previously had a cancer or a malignant tumour) + 0 points (maximum score for memory) + 10 points (never had a chance to do things that he never experienced before) + 5 points (difficulties doing work around house and garden) + 14 points (struggling to walk 100 yards) + 19 points (describing health as poor) + 0 points (having never experienced stroke) + 0 points (no history of chronic lung disease) = 164. This corresponds to a normalized prognostic index of 1.69 (linear predictor line) for all-cause mortality, meaning that the participant has a probability to die in the following 10 years in the range 35.68–62.48%.	
A more precise way to compute the probabilities of death during the next 10 years for this patient is to use the following formula, as presented in Additional file 14, for absolute risk predictions at time *t*:	
$$ 1-{S}_0{(t)}^{\exp \left({b}_1{x}_1+{b}_2{x}_2+{b}_3{x}_3+\dots \right)} $$,	
where *S*_0_(*t*) is the baseline survival probability at time *t*, *x*_*i*_ are the variables and *b*_*i*_ are the log hazard ratios, i.e. the Cox-Lasso estimated coefficients (Table 2).	
Therefore, given *S*_0_(*t*) = 0.9985, for the same individual as above, the probability of death during the next 10 years will be precisely 57.19%:	
1 − 0.9985^exp(0.0695 × 75 + 0.1787 × 1 − 0.0116 × 0 + 0.0532 × 1 + 0.0435 × 0 + 0.0316 × 1 + 0.0686 × 1 + 0.0509 × 0 + 0.0861 × 1 + 0.1198 × 1 + 0.2498 × 1 + 0.3369 × 1 + 0.3158 × 0)^ = 0.5719 = 57.19%	

### External validation

When applied to the validation sample, our model demonstrated good discrimination (*c*-index=0.69) (Table 1). The distributions of prognostic index estimated based on 13 variables included in the model in the development cohort and external cohort closely aligned (Additional file 11). Normalized PI highlighted that probability of death increased linearly with the higher quantiles with those scoring at the top 10% quantile having 62.5% probability of dying in the following 10 years (Table 4). The frequency of predicted survival probabilities closely aligned in the derivation and the validation cohorts (Additional file 11). Calibration plot showed an overall good agreement between the predicted and observed survival rates at 10-years as estimated by our newly developed model (Fig. 2). The calibration of our final model improved once it was externally validated; although it was slightly under 1 (calibration slopes *β*=0.80), it was higher compared to the calibration slope of the externally validated 10-item index calibration slope *β*=0.75). When further compared to our model, the externally validated 10-item index had higher discrimination (*c*-index=0.75); its estimated sensitivity (73.7%) was comparable to our model (71.5%), but specificity was considerably lower (64.4%) when compared with specificity of our externally validated model (70.6%). The mean predicted risk of all-cause mortality based on our model was 4.39% (SD_internal_=0.63) in the derivation sample and 8.51% (SD_external_=0.89) in the validation sample.

**Table 4:** Normalized prognostic indexes (PI) for mortality translated into probabilities of all-cause mortality during a 10-year follow-up period

Quantile	Normalized PI for mortality	Probability (%) of death at 10 years
0%	−1.12	3.88
10%	−0.79	5.37
20%	−0.62	6.31
30%	−0.43	7.54
40%	−0.25	9.01
50%	−0.04	10.94
60%	0.14	13.04
70%	0.35	15.79
80%	0.58	19.48
90%	0.84	24.56
100%	2.09	62.48

## Discussion

Utilising methods advocated for clinical and personalised risk prediction [13], we developed, evaluated and validated a prediction model for estimating an individual risk of all-cause mortality in the following 10 years. The model is calibrated for individuals aged 50–75 years living in England but generalises reasonably well to other populations with similar underlying characteristics. It also has good sensitivity and specificity reducing unnecessary testing without potentially compromising need for care [38]. From an extensive pool of 84 factors, the model identified 13 prognostic variables and quantified their predictive importance for 10-year mortality risk for older adults in the general population. Based on an individual profile of risk factors, our model allows a rapid assessment of an individual risk for all-cause mortality, which is at the core of more in-depth risk assessment, follow-up monitoring and individually tailored prevention strategies [39]. Therefore, it can be used as a first-stage screening aid that might prolong life-expectancy by alerting to an individual’s heightened risk profile and a need for more targeted evaluation and prevention. It could also be used by non-professionals to improve self-awareness of their health status, and by governmental and health organisations to decrease the burden of certain risk factors in the general population of older people.

Identification of the most robust predictors of all-cause mortality is pivotal for efficient early interventions and prevention services. Around 50% of the prognostic factors identified by our model overlapped with those included in the 10-item index [7]. These included age, current smoking, diagnosis of cancer, history of chronic lung disease, difficulty walking 100 yards and male gender. Because RRMs simultaneously handle a large number of variables in a statistically correct way [8] while avoiding overfitting, unlike the 10-item index [7] and prognostic indexes that predated it [4–6], we were able to identity novel potential prognostic factors for all-cause mortality in older adults. These included lower accumulated wealth, lack of self-realisation, poor self-rated health and lower working memory. This may suggest that less wealthy may be more subject to poor physical and social environments, which can encourage health-damaging behaviours [40], leading to premature death. The lack of self-realisation, which reflects a reduced sense of purpose in life, has also been linked to decreased longevity [41]. Consequently, the consideration of these factors will help identify high-risk groups who might otherwise be under-detected.

Several prognostic factors included in the 10-item index [7], such as history of heart failure, no vigorous physical activity and mobility difficulties related to pulling/pushing large objects and preparing meals, were not selected by our model. In agreement with our results, the mortality index developed in US [5] on which the 10-item index [7] was modelled, did not include sedentary lifestyle and difficulty preparing meals as risk factors for mortality in older people. Thus, these two factors might have been mere proxies for other unmeasured variables related to all-cause mortality in elderly. Not selecting prior history of heart failure by our model, even though this variable was included in previous mortality indexes [4–7], may be explained by the methodological properties inherent to RRMs. As a means of identifying true prognostic factors from a pool of possibly related variables, RRMs omit unnecessary variables through the introduction of a penalty, which shrinks the correlated variables towards zero [24, 25]. It is feasible, therefore, that this variable was omitted due to the presence of other related variables, such as self-rated health, which has been linked to mortality through its association with cardiovascular diseases [42]. Elastic net approach may be preferred if a set of all correlated variables should be either included in a model at the cost of retaining a larger number of variables or excluded altogether [43].

External validation is essential to ensure the quality of the prediction model and its potential usefulness in clinical practice. Although our model showed good externally validated discrimination, the finding which was unlikely to be due to chance [19, 44], the externally validated discrimination of the 10-item index [7] was higher compared with our model. This is consistent with some recent studies, which showed that statistical learning methods, including machine learning algorithms, lead to only limited, if any [45], incremental improvements in models’ performances [46, 47] when compared with simpler statistical methods if such model building was theory-driven [48]. Nonetheless, compared with our model, the 10-item index [7], based on prognostic factors chosen through multiple sequential hypothesis testing, was more likely to overfit the data. Specificity of the externally validated 10-item index was also considerably lower (64.4%) compared to our externally validated model (70.5%), implying it is likely to falsely classify a higher proportion of older adults as high risk for all-cause mortality in the following 10 years.

### Strengths and weaknesses

Our prediction algorithm has several advantages. The model is based on absolute risks determined and validated in two very large and independent populations. We employed rigorous methods for model building and validation when accuracy, interpretability and parsimony are the priority, following the recommended guidelines of model building and reporting. To avoid using unrepresentative sample of complete cases that may result in incorrect risk predictions [49, 50], we catered for missing values. The cohorts employed for model development and validation were representative of older people resigning in England and US. The items included in our model are often collected in epidemiological studies and are ascertainable during a brief patient-physician discussion. This is also the first study to provide measures of prediction accuracy of an existing all-cause mortality model as measured in an external sample. Nonetheless, as with the 10-item index [7], our model may not be applicable to nursing home and other institutional populations. Our model was developed and validated in the cohorts with predominately white ethnicity. Although this is consistent with a wide range of prediction models for health-related outcomes [1–3, 51], further validation in more ethnically diverse populations is required. As with many risk models, we only accounted for baseline variables, although for many time-varying factors, exposure status may change during the follow-up period [52]. However, using baseline variables reflects the real-life clinical information available to a physician and a participant when they need to make decisions on the likely risk of all-cause mortality for an individual during the next 10 years. Finally, it would be of interest to include potential interaction with a smaller set of candidate predictors in the future studies.

## Conclusion

Having employed modern statistical learning algorithms and addressed the weaknesses of previous models, a new mortality model achieved good discrimination and calibration as shown by its performance in a separate validation cohort. Our model relies on 13 variables, which are available by patient report in a variety of care and research settings. It allows rapid estimations of an individual’s risk of all-cause mortality based on an individual risk profile. These characteristics suggest that our model may be useful for clinical, policy, and epidemiological applications.

## Supplementary Information

**Additional file 1.** Outlines a list of all variables considered in the analyses and whether they have been included or excluded from the model building.

**Additional file 2.** Distribution of missing and observed variables included in the analyses in ELSA.

**Additional file 3.** Sample calculations for Survival outcomes (Cox prediction models).

**Additional file 4.** Distributions of the variables at baseline before and after multiple imputations.

**Additional file 5.** Apparent coefficients for the Cox-LASSO regression for all-cause mortality during the 10-year follow-up.

**Additional file 6.** Estimated optimism.

**Additional file 7.** Apparent models’ performance in prediction the 10-year risk of all-cause mortality in older adults.

**Additional file 8.** Optimism-corrected models’ performance in prediction the 10-year risk of all-cause mortality in older adults.

**Additional file 9.** Apparent models’ discrimination in prediction the 10-year risk of all-cause mortality in older adults.

**Additional file 10.** Internally validated though optimism-correction models’ discrimination for prediction the 10-year risk of all-cause mortality in older adults.

**Additional file 11.** Histogram depicting distribution of prognostic index (PI) estimated based on 13 variables included in the model in the development cohort and external cohort.

**Additional file 12.** The distribution of survival probabilities estimated based on 13 variables included in the model in the development and validation cohorts.

**Additional file 13.** Distributions of the variables included in the final all-cause mortality model in derivation cohort (ELSA) and validation cohort (HRS).

**Additional file 14.** The formula for the final model.

## Data Availability

The English Longitudinal Study of Ageing (ELSA) was developed by a team of researchers based at University College London, the Institute for Fiscal Studies and the National Centre for Social Research. The datasets generated and/or analysed during the current study are available in UK Data Services and can be accessed at: https://discover.ukdataservice.ac.uk. No administrative permissions were required to access these data.

## Abbreviations

ELSA: English Longitudinal Study of Ageing
HRS: Health and Retirement Study
RRMs: regularised regression methods
TRIPOD: Transparent Reporting of a multivariable prediction model for Individual Prognosis Or Diagnosis guidelines
CV: Cross-validation
ROC: Receiver operating characteristic curve
SD: Standard deviation
US: United States
Cox-Lasso: Cox proportional hazards model with regularisation by the least absolute shrinkage and selection operator
