# Supplementary material for: Development and validation of prediction model to estimate 10-year risk of all-cause mortality using modern statistical learning methods: a large population-based cohort study and external validation
Source: BMC Med Res Methodol. 2021 Jan 6;21:8. doi: 10.1186/s12874-020-01204-7 (PMC7789636; doi:10.1186/s12874-020-01204-7)
Supplement: Supplementary file 1 — Additional file 1. Outlines a list of all variables considered in the analyses and whether they have been included or excluded from the model building. [file 12874_2020_1204_MOESM1_ESM.docx]

**Additional file 1. Outlines a list of all variables considered in the analyses and whether they have been included or excluded from the model building.**

| **List of predictors included in the analyses** | **Included in the models?** |  | **List of predictors included in the analyses** | **Included in the models?** |
| --- | --- | --- | --- | --- |
| Age (years) | Y |  | Difficulty pulling or pushing large objects | Y |
| Arthritis | Y |  | Difficulty reaching or extending arms above shoulder level | Y |
| Body Mass Index | Y |  | Difficulty shopping for groceries | Y |
| Cancer | Y |  | Difficulty sitting 2 hours | Y |
| Chronic Heart Disease | Y |  | Difficulty stooping, kneeling or crouching | Y |
| CASP: My age prevents me from doing the things I would like to | Y |  | Difficulty taking medications | Y |
| CASP: I feel that what happens to me is out of my control | Y |  | Difficulty using map to figure out how to get around strange place | Y |
| CASP: I never feel free to plan for the future | Y |  | Difficulty using the toilet, including getting up or down | Y |
| CASP: I feel left out of things | Y |  | Difficulty walking 100 yards | Y |
| CASP: I cannot do the things I want to do | Y |  | Difficulty walking across a room | Y |
| CASP: Family responsibilities prevent me from doing what I want to do | Y |  | Do you find it difficult to follow a conversation | Y |
| CASP: I do not feel that I can please myself with what I do | Y |  | Fair self-rated memory | Y |
| CASP: Shortage of money stops me from doing the things I want to do | N |  | History of dementia | N |
| CASP: I never feel that my life has meaning | Y |  | Have you ever had cataract surgery? | N |
| CASP: I never enjoy the things that I do | N |  | Has/ve children | Y |
| CASP: I never enjoy being in the company of others | N |  | Have you ever fractured your hip? | Y |
| CASP: My health stops me from doing things I want to do | Y |  | Have you fallen down in the last two years (for any reason)? | Y |
| CASP: I never feel full of energy these days | Y |  | How many times have you fallen down in the last two years? | N |
| CASP: I never choose to do things that I have never done before | Y |  | Job status: Professional | Y |
| CASP: I never feel satisfied with the way my life has turned out | Y |  | Job status: Skilled manual | Y |
| CASP: I never feel that life is full of opportunities | Y |  | Job status: Skilled non-manual | Y |
| CASP: I never feel that the future looks good to me | N |  | Job status: Unskilled | Y |
| CASP: I do not look forward to each day | N |  | Hypertension | Y |
| Chronic Lung Disease | Y |  | In [that fall/any of the falls] did you injure yourself seriously enough to need | N |
| Cognition: Executive function | Y |  | Limiting longstanding illness any | Y |
| Cognition: fluency | Y |  | Lives in urban areas | Y |
| Cognition: memory | Y |  | Living alone | Y |
| Cognition: orientation | Y |  | Low level of wealth | Y |
| Cognition: Processing speed | Y |  | Male gender | Y |
| Currently a smoker | Y |  | Mortality | Y |
| Currently unemployed | Y |  | No close friends | Y |
| Daily alcohol use | Y |  | No qualification | Y |
| Depression | Y |  | No vigorous/moderate activity at least once per week | Y |
| Diabetes | Y |  | Not in a relationship | Y |
| Difficulty bathing or showering | Y |  | Not involved in any organisations | Y |
| Difficulty climbing one flight stairs without resting | Y |  | Number of friends 1 or less | Y |
| Difficulty climbing several flights stairs without resting | Y |  | Number of mobility impairments | Y |
| Difficulty doing work around house and garden | Y |  | Owns own house | Y |
| Difficulty dressing, including putting on shoes and socks | Y |  | Poor eyesight | Y |
| Difficulty eating, such as cutting up food | Y |  | Poor eyesight for seeing things at a distance | Y |
| Difficulty getting in and out of bed | Y |  | Poor eyesight for seeing things up close | Y |
| Difficulty getting up from chair after sitting long periods | Y |  | Poor hearing | Y |
| Difficulty lifting or carrying weights over 10 pounds | Y |  | Poor self-rated health | Y |
| Difficulty making telephone calls | Y |  | Poor self-rated memory | Y |
| Difficulty managing money, eg paying bills, keeping track expenses | Y |  | Social isolation excluding marriage | Y |
| Difficulty picking up 5p coin from table | Y |  | Stroke | Y |
| Difficulty preparing a hot meal | Y |  | Survival time | Y |
| CASP: I never look back on my life with a sense of happiness | N |  | White ethnicity | Y |

CASP, Quality of Life Scale (CASP-19)
