# Supplementary material for: Development and validation of prediction model to estimate 10-year risk of all-cause mortality using modern statistical learning methods: a large population-based cohort study and external validation
Source: BMC Med Res Methodol. 2021 Jan 6;21:8. doi: 10.1186/s12874-020-01204-7 (PMC7789636; doi:10.1186/s12874-020-01204-7)
Supplement: Supplementary file 2 — Additional file 2. Distribution of missing and observed variables included in the analyses in ELSA. [file 12874_2020_1204_MOESM2_ESM.docx]

**Additional file 2. Distribution of missing and observed variables included in the analyses in ELSA**

| **Variables** | **N**  **missing** | **% missing** | **N complete** | **%**  **complete** |
| --- | --- | --- | --- | --- |
| Age (years) | 0 | 0.0 | 9154 | 100.0 |
| Arthritis | 4316 | 47.1 | 4838 | 52.9 |
| Body Mass Index | 2477 | 27.1 | 6677 | 72.9 |
| Cancer | 0 | 0.0 | 9154 | 100.0 |
| CASP: Family responsibilities prevent me from doing what I want to do | 848 | 9.3 | 8306 | 90.7 |
| CASP: I cannot do the things I want to do | 833 | 9.1 | 8321 | 90.9 |
| CASP: I never choose to do things that I have never done before | 842 | 9.2 | 8312 | 90.8 |
| CASP: I never enjoy the things that I do | 777 | 8.5 | 8377 | 91.5 |
| CASP: I never feel free to plan for the future | 938 | 10.2 | 8216 | 89.8 |
| CASP: I never feel full of energy these days | 803 | 8.8 | 8351 | 91.2 |
| CASP: I never feel satisfied with the way my life has turned out | 828 | 9.0 | 8326 | 91 |
| CASP: I never feel that I can please myself with what I do | 831 | 9.1 | 8323 | 90.9 |
| CASP: I never feel that life is full of opportunities | 838 | 9.1 | 8316 | 90.9 |
| CASP: I never feel that my life has meaning | 839 | 9.2 | 8315 | 90.8 |
| CASP: I never feel that the future looks good to me | 823 | 9.0 | 8331 | 91 |
| CASP: I never look back on my life with a sense of happiness | 792 | 8.6 | 8362 | 91.4 |
| CASP: My age prevents me from doing the things I would like to | 808 | 8.8 | 8346 | 91.2 |
| CASP: My health stops me from doing things I want to do | 809 | 8.8 | 8345 | 91.2 |
| CASP: Shortage of money stops me from doing the things I want to do | 816 | 8.9 | 8338 | 91.1 |
| CASP: I feel left out of things | 871 | 9.5 | 8283 | 90.5 |
| CASP: I feel that what happens to me is out of my control | 890 | 9.7 | 8264 | 90.3 |
| Chronic Heart Disease | 1585 | 17.3 | 7569 | 82.7 |
| Chronic Lung Disease | 4316 | 47.1 | 4838 | 52.9 |
| Cognition: Executive function | 230 | 2.5 | 8924 | 97.5 |
| Cognition: fluency | 230 | 2.5 | 8924 | 97.5 |
| Cognition: memory | 224 | 2.4 | 8930 | 97.6 |
| Cognition: orientation | 116 | 1.3 | 9038 | 98.7 |
| Cognition: Processing speed | 452 | 4.9 | 8702 | 95.1 |
| Currently a smoker | 3337 | 36.5 | 5817 | 63.5 |
| Currently unemployed | 0 | 0.0 | 9154 | 100.0 |
| Daily alcohol use | 99 | 1.1 | 9055 | 98.9 |
| Depression | 282 | 3.1 | 8872 | 96.9 |
| Diabetes | 0 | 0.0 | 9154 | 100.0 |
| Difficulty bathing or showering | 85 | 0.9 | 9069 | 99.1 |
| Difficulty climbing one flight stairs without resting | 98 | 1.1 | 9056 | 98.9 |
| Difficulty climbing several flights stairs without resting | 98 | 1.1 | 9056 | 98.9 |
| Difficulty doing work around house and garden | 85 | 0.9 | 9069 | 99.1 |
| Difficulty dressing, including putting on shoes and socks | 98 | 1.1 | 9056 | 98.9 |
| Difficulty eating, such as cutting up food | 85 | 0.9 | 9069 | 99.1 |
| Difficulty getting in and out of bed | 85 | 0.9 | 9069 | 99.1 |
| Difficulty getting up from chair after sitting long periods | 98 | 1.1 | 9056 | 98.9 |
| Difficulty lifting or carrying weights over 10 pounds | 98 | 1.1 | 9056 | 98.9 |
| Difficulty making telephone calls | 85 | 0.9 | 9069 | 99.1 |
| Difficulty managing money, eg paying bills, keeping track expenses | 85 | 0.9 | 9069 | 99.1 |
| Difficulty picking up 5p coin from table | 98 | 1.1 | 9056 | 98.9 |
| Difficulty preparing a hot meal | 85 | 0.9 | 9069 | 99.1 |
| Difficulty pulling or pushing large objects | 98 | 1.1 | 9056 | 98.9 |
| Difficulty reaching or extending arms above shoulder level | 98 | 1.1 | 9056 | 98.9 |
| Difficulty shopping for groceries | 85 | 0.9 | 9069 | 99.1 |
| Difficulty sitting 2 hours | 98 | 1.1 | 9056 | 98.9 |
| Difficulty stooping, kneeling or crouching | 98 | 1.1 | 9056 | 98.9 |
| Difficulty taking medications | 85 | 0.9 | 9069 | 99.1 |
| Difficulty using map to figure out how to get around strange place | 85 | 0.9 | 9069 | 99.1 |
| Difficulty using the toilet, including getting up or down | 85 | 0.9 | 9069 | 99.1 |
| Difficulty walking 100 yards | 98 | 1.1 | 9056 | 98.9 |
| Difficulty walking across a room | 85 | 0.9 | 9069 | 99.1 |
| Do you find it difficult to follow a conversation | 101 | 1.1 | 9053 | 98.9 |
| Fair self-rated memory | 193 | 2.1 | 8961 | 97.9 |
| Has/ve children | 765 | 8.4 | 8389 | 91.6 |
| Have you ever fractured your hip? | 4381 | 47.9 | 4773 | 52.1 |
| Have you fallen down in the last two years (for any reason)? | 4382 | 47.9 | 4772 | 52.1 |
| Job status: Professional | 338 | 3.7 | 8816 | 96.3 |
| Job status: Skilled manual | 338 | 3.7 | 8816 | 96.3 |
| Job status: Skilled non-manual | 338 | 3.7 | 8816 | 96.3 |
| Job status: Unskilled | 338 | 3.7 | 8816 | 96.3 |
| Hypertension | 0 | 0.0 | 9154 | 100.0 |
| Limiting longstanding illness any | 9 | 0.1 | 9145 | 99.9 |
| Lives in urban areas | 2 | 0.0 | 9152 | 100.0 |
| Living alone | 0 | 0.0 | 9154 | 100.0 |
| Low level of wealth | 709 | 7.7 | 8445 | 92.3 |
| Male gender | 0 | 0.0 | 9154 | 100.0 |
| Mortality | 0 | 0.0 | 9154 | 100.0 |
| No close friends | 754 | 8.2 | 8400 | 91.8 |
| No qualification | 809 | 8.8 | 8345 | 91.2 |
| No vigorous/moderate activity at least once per week | 1585 | 17.3 | 7569 | 82.7 |
| Not in a relationship | 2 | 0.0 | 9152 | 100.0 |
| Not involved in any organisations | 1004 | 11.0 | 8150 | 89.0 |
| Number of friends 1 or less | 1686 | 18.4 | 7468 | 81.6 |
| Number of mobility impairments | 98 | 1.1 | 9056 | 98.9 |
| Owns own house | 40 | 0.4 | 9114 | 99.6 |
| Poor eyesight | 7 | 0.1 | 9147 | 99.9 |
| Poor eyesight for seeing things at a distance | 119 | 1.3 | 9035 | 98.7 |
| Poor eyesight for seeing things up close | 120 | 1.3 | 9034 | 98.7 |
| Poor hearing | 9 | 0.1 | 9145 | 99.9 |
| Poor self-rated health | 101 | 1.1 | 9053 | 98.9 |
| Poor self-rated memory | 193 | 2.1 | 8961 | 97.9 |
| Social isolation excluding marriage | 754 | 8.2 | 8400 | 91.8 |
| Stroke | 0 | 0.0 | 9154 | 100.0 |
| Survival time | 1791 | 19.6 | 7363 | 80.4 |
| White ethnicity | 60 | 0.7 | 9094 | 99.3 |

CASP, Quality of Life Scale (CASP-19)
