# Supplementary material for: Development and validation of prediction model to estimate 10-year risk of all-cause mortality using modern statistical learning methods: a large population-based cohort study and external validation
Source: BMC Med Res Methodol. 2021 Jan 6;21:8. doi: 10.1186/s12874-020-01204-7 (PMC7789636; doi:10.1186/s12874-020-01204-7)
Supplement: Supplementary file 3 — Additional file 3. Sample calculations for Survival outcomes (Cox prediction models). [file 12874_2020_1204_MOESM3_ESM.docx]

**Additional file 3.** Sample calculations for Survival outcomes (Cox prediction models)

Information necessary to calculate sample size:

  1) the anticipated outcome event rate

  ) the number of candidate predictor parameters

  3) the anticipated mean follow-up years

  4) the anticipated Cox-Snell adjusted R squared statistic (R2cs)

  5) specify a time point of interest

1) The total person-years 119037; 1240 people have the outcome of interest

rate<-1240/119037

2) The number of candidate predictor parameters

predictors <- 84

3) The is the total sample:

n<-9154

The length of follow-up is measured in years:

mean_fu_yrs<-143.4212/12

The total person-years follow-up; follow up is measured in years

personTimeFU_yrs<-mean_fu_yrs*n

The anticipated outcome event rate:

expectedEvents<-personTimeFU_yrs*rate

Standardise as recommended in the paper (page 5 of supplementary materials):

rate_st=rate*personTimeFU_yrs/n

(lnLnull_std= (rate_st * n * ln(rate_st))-(rate_st * n))

The maximum possible R-squared as defined by equation 23 (Riley et al. 2018):

(maxR2cs_std<-1-exp(2*lnLnull_std/n))

Calculating sample size

pmsampsize(type = "s", rsquared = 0.15*maxR2cs_std, parameters = predictors, rate = rate,timepoint = 10, meanfup = mean_fu_yrs)

NB: Assuming 0.05 acceptable difference in apparent & adjusted R-squared

NB: Assuming 0.05 margin of error in estimation of overall risk at time point = 10

NB: Events per Predictor Parameter (EPP) assumes overall event rate = 0.01041693

Samp_size Shrinkage Parameter Rsq Max_Rsq EPP

Criteria 1 8978 0.900 84 0.08039382 0.54 13.31

Criteria 2 2948 0.749 84 0.08039382 0.54 4.37

Criteria 3 * 8978 0.900 84 0.08039382 0.54 13.31

Final 8978 0.900 84 0.08039382 0.54 13.31

Minimum sample size required for new model development based on user inputs = 8978,

corresponding to 107303 person-time** of follow-up, with 1118 outcome events

assuming an overall event rate = 0.01041693 and therefore an EPP = 13.31

* 95% CI for overall risk = (0.093, 0.104), for true value of 0.099 and sample size n = 8978

**where time is in the units mean follow-up time was specified in
