# Supplementary material for: Development and validation of prediction model to estimate 10-year risk of all-cause mortality using modern statistical learning methods: a large population-based cohort study and external validation
Source: BMC Med Res Methodol. 2021 Jan 6;21:8. doi: 10.1186/s12874-020-01204-7 (PMC7789636; doi:10.1186/s12874-020-01204-7)
Supplement: Supplementary file 4 — Additional file 4. Distributions of the variables at baseline before and after multiple imputations. [file 12874_2020_1204_MOESM4_ESM.docx]

**Additional file 4. Distributions of the variables at baseline before and after multiple imputations**

| **Variables** | **Variables before imputation** | **Variables after imputation** |
| --- | --- | --- |
|  | **n (%) / mean (SD)** | **n (%) / mean (SD)** |
| Age (years) | 61.5 (7.2) | 61.5 (7.2) |
| Arthritis | 2156 (44.6) | 3505 (38.3) |
| Body Mass Index | 28.1 (4.9) | 28.2 (4.3) |
| Cancer | 521 (5.7) | 521 (5.7) |
| CASP: Family responsibilities prevent me from doing what I want to do | 5222 (62.9) | 5935 (64.8) |
| CASP: I cannot do the things I want to do | 385 (4.6) | 386 (4.2) |
| CASP: I do not feel that I can please myself with what I do | 344 (4.1) | 346 (3.8) |
| CASP: I feel left out of things | 3893 (47.0) | 3953 (43.2) |
| CASP: I feel that what happens to me is out of my control | 3552 (43.0) | 3614 (39.5) |
| CASP: I never choose to do things that I have never done before | 787 (9.5) | 818 (8.9) |
| CASP: I never feel free to plan for the future | 765 (9.3) | 775 (8.5) |
| CASP: I never feel full of energy these days | 420 (5.9) | 446 (4.9) |
| CASP: I never feel satisfied with the way my life has turned out | 268 (3.2) | 270 (3.0) |
| CASP: I never feel that life is full of opportunities | 311 (3.7) | 313 (3.4) |
| CASP: I never feel that my life has meaning | 8106 (97.5) | 8943 (97.7) |
| CASP: I never feel that the future looks good to me | 287 (3.4) | 290 (3.2) |
| CASP: My age prevents me from doing the things I would like to | 2880 (34.5) | 2926 (32.0) |
| CASP: My health stops me from doing things I want to do | 4807 (57.6) | 5555 (60.7) |
| CASP: Shortage of money stops me from doing the things I want to do | 6369 (76.3) | 7161 (78.2) |
| Chronic Heart Disease | 757 (10.0) | 791 (8.6) |
| Chronic Lung Disease | 515 (10.6) | 533 (5.8) |
| Cognition: executive function | 20.1 (6.2) | 20.0 (6.2) |
| Cognition: fluency | 20.1 (6.2) | 20.0 6.2) |
| Cognition: memory | 10.0 (3.3) | 9.9 (3.3) |
| Cognition: orientation | 3.7 (0.6) | 3.7 (9.6) |
| Cognition: processing speed | 14.2 (9.1) | 14.3 (8.9 |
| Currently a smoker | 1812 (31.1) | 1812 (31.1) |
| Currently unemployed | 1528 (16.7) | 1528 (16.7) |
| Daily alcohol use | 2597 (28.7) | 2597 (28.7) |
| Depression | 1361 (15.3) | 1361 (15.3) |
| Diabetes | 636 (6.9) | 636 (6.9) |
| Difficulty bathing or showering | 842 (9.3) | 845 (9.2) |
| Difficulty climbing one flight stairs without resting | 1073 (11.8) | 1073 (11.8) |
| Difficulty climbing several flights stairs without resting | 2933 (32.4) | 2933 (32.4) |
| Difficulty doing work around house and garden | 1164 (12.8) | 1172 (12.8) |
| Difficulty dressing including putting on shoes and socks | 1048 (11.6) | 1055 (11.5) |
| Difficulty eating such as cutting up food | 146 (1.6) | 146 (1.6) |
| Difficulty getting in and out of bed | 551 (6.1) | 554 (6.0) |
| Difficulty getting up from chair after sitting long periods | 2144 (23.7) | 2144 (23.7) |
| Difficulty lifting or carrying weights over 10 pounds | 1989 (22.0) | 1989 (22.0) |
| Difficulty making telephone calls | 103 (1.1) | 103 (1.1) |
| Difficulty managing money eg paying bills, keeping track expenses | 157 (1.7) | 157 (1.7) |
| Difficulty picking up 5p coin from table | 380 (4.20) | 380 (4.2) |
| Difficulty preparing a hot meal | 311 (3.4) | 312 (3.4) |
| Difficulty pulling or pushing large objects | 1386 (15.3) | 1386 (15.3) |
| Difficulty reaching or extending arms above shoulder level | 920 (10.2) | 920 1(0.2) |
| Difficulty shopping for groceries | 632 (7.0) | 635 (6.9) |
| Difficulty sitting 2 hours | 1279 (14.1) | 1279 (14.1) |
| Difficulty stooping kneeling or crouching | 2907 (32.1) | 2907 (32.1) |
| Difficulty taking medications | 121 (1.3) | 121 (1.3) |
| Difficulty using map to figure out how to get around strange place | 377 (4.2) | 377 (4.12) |
| Difficulty using the toilet including getting up or down | 272 (3.0) | 273 (3.0) |
| Difficulty walking 100 yards | 887 (9.8) | 887 (9.8) |
| Difficulty walking across a room | 225 (2.5) | 226 (2.5) |
| Do you find it difficult to follow a conversation | 2869 (31.7) | 2869 (31.7) |
| Fair self-rated memory | 2296 (25.6) | 2296 (25.6) |
| Has/ve children | 7364 (87.8) | 7364 (87.8) |
| Have you ever fractured your hip? | 49 (1.0) | 49 (1.0) |
| Have you fallen down in the last two years (for any reason)? | 1291 (27.0) | 1291 (27.0) |
| Job status: Professional | 2919 (33.1) | 2919 (33.1) |
| Job status: Skilled manual | 2046 (23.2) | 2046 (23.2) |
| Job status: Skilled non-manual | 3213 (36.4) | 3213 (36.4) |
| Job status: Unskilled | 638 (7.2) | 638 (7.24) |
| Hypertension | 3313 (36.2) | 3313 (36.2) |
| Limiting longstanding illness any | 2945 (32.2 | 2949 (32.2) |
| Lives in urban areas | 6839 (74.7) | 6841 (74.7) |
| Living alone | 1766 (19.3) | 1766 (19.3) |
| Low level of wealth | 2513 (29.8) | 3113 (34.0) |
| Male gender | 4263 (46.6) | 4263 (46.6) |
| Mortality | 1270 (13.9) | 1270 (13.9) |
| No close friends | 495 (5.9) | 500 (5.5) |
| No qualification | 3497 (41.9) | 3907 (42.7) |
| No vigorous/moderate activity at least once per week | 2691 (35.5) | 3353 (36.6) |
| Not in a relationship | 1615 (17.6) | 1615 (17.6) |
| Not involved in any organisations | 2416 (29.6) | 2814 (30.7) |
| Number of friends 1 or less | 2211 (29.6) | 2570 (28.1) |
| Number of mobility impairments | 1.7 (2.4) | 1.8 (2.4) |
| Owns own house | 7510 (82.4) | 7545 (82.4) |
| Poor eyesight | 1615 (17.6) | 1225 (13.4) |
| Poor eyesight for seeing things at a distance | 656 (7.3) | 688 (7.5) |
| Poor eyesight for seeing things up close | 889 (9.8) | 921 (10.1) |
| Poor hearing | 1723 (18.8) | 1725 (18.8) |
| Poor self-rated health | 2252 (24.9) | 2299 (25.1) |
| Poor self-rated memory | 520 (5.8) | 522 (5.7) |
| Social isolation excluding marriage | 687 (8.2) | 687 (7.5) |
| Stroke | 294 (3.2) | 294 (3.2) |
| Survival time (months) | 167.2 (43.0) | 167.2 (43.0) |
| White ethnicity | 8796 (96.7) | 8854 (96.7) |

CASP, Quality of Life Scale (CASP-19); SD, standard deviation
