# Supplementary material for: Development and validation of prediction model to estimate 10-year risk of all-cause mortality using modern statistical learning methods: a large population-based cohort study and external validation
Source: BMC Med Res Methodol. 2021 Jan 6;21:8. doi: 10.1186/s12874-020-01204-7 (PMC7789636; doi:10.1186/s12874-020-01204-7)
Supplement: Supplementary file 5 — Additional file 5. Apparent coefficients for the Cox-LASSO regression for all-cause mortality during the 10-year follow-up. [file 12874_2020_1204_MOESM5_ESM.docx]

**Additional file 5. Apparent coefficients for the Cox-LASSO regression for all-cause mortality during the 10-year follow-up.**

| **Predictor variables** | **Model_Best_** | **Model_1-SE_** | **Model_3%_** |
| --- | --- | --- | --- |
| Age (years) | 0.10 | 0.10 | 0.07 |
| Arthritis (“yes”=1) | -0.18 | -0.17 |  |
| BMI | -0.01 | -0.01 |  |
| Cancer | 0.71 | 0.70 | 0.12 |
| CASP: Family responsibilities prevent me from doing what I want to do (“yes”=1) | 0.02 | 0.02 |  |
| CASP: I cannot do the things I want to do (“yes”=1) |  |  |  |
| CASP: I never choose to do things that I have never done before (“yes”=1) | 0.29 | 0.29 | 0.18 |
| CASP: I never feel free to plan for the future (“yes”=1) |  |  |  |
| CASP: I never feel full of energy these days (“yes”=1) | 0.05 | 0.04 |  |
| CASP: I feel left out of things (“yes”=1) |  |  |  |
| CASP: I never feel satisfied with the way my life has turned out (“yes”=1) |  |  |  |
| CASP: I never feel that I can please myself what I do (“yes”=1) |  |  |  |
| CASP: I never feel that life is full of opportunities (“yes”=1) |  |  |  |
| CASP: I never feel that my life has meaning (“yes”=1) |  |  |  |
| CASP: I feel that what happens to me is out of my control (“yes”=1) | -0.01 | -0.002 |  |
| CASP: My age prevents me from doing the things I would like to (“yes”=1) | -0.04 | -0.03 |  |
| CASP: My health stops me from doing things I want to do (“yes”=1) | -0.09 | -0.08 |  |
| Chronic Heart Disease (“yes”=1) |  |  |  |
| Chronic lung disease (“yes”=1) | 0.43 | 0.43 | 0.32 |
| Cognition: Executive function | 0.00 | -0.001 |  |
| Cognition: Fluency |  |  |  |
| Cognition: Memory | -0.03 | -0.03 | -0.01 |
| Cognition: Orientation | -0.08 | -0.08 |  |
| Cognition: Processing speed | 0.00 | 0.001 |  |
| Currently a smoker (“yes”=1) | 0.45 | 0.45 | 0.07 |
| Currently unemployed (“yes”=1) | 0.10 | 0.09 |  |
| Daily alcohol use (“yes”=1) | 0.05 | 0.04 |  |
| Depression (“yes”=1) |  |  |  |
| Diabetes (“yes”=1) | 0.19 | 0.18 |  |
| Difficulty bathing or showering (“yes”=1) |  |  |  |
| Difficulty climbing one flight stairs without resting (“yes”=1) |  |  |  |
| Difficulty climbing several flights stairs without resting (“yes”=1) | 0.19 | 0.18 |  |
| Difficulty doing work around house and garden (“yes”=1) | 0.17 | 0.16 | 0.09 |
| Difficulty dressing, including putting on shoes and socks (“yes”=1) |  |  |  |
| Difficulty eating, such as cutting up food (“yes”=1) | 0.50 | 0.48 |  |
| Difficulty getting in and out of bed (“yes”=1) | -0.08 | -0.06 |  |
| Difficulty getting up from chair after sitting long periods (“yes”=1) |  |  |  |
| Difficulty lifting or carrying weights over 10 pounds (“yes”=1) |  |  |  |
| Difficulty making telephone calls (“yes”=1) | 0.24 | 0.24 |  |
| Difficulty managing money, eg paying bills, keeping track expenses (“yes”=1) | 0.01 | 0.01 |  |
| Difficulty picking up 5p coin from table (“yes”=1) |  |  |  |
| Difficulty preparing a hot meal (“yes”=1) |  |  |  |
| Difficulty pulling or pushing large objects (“yes”=1) |  |  |  |
| Difficulty reaching or extending arms above shoulder level (“yes”=1) | -0.10 | -0.08 |  |
| Difficulty shopping for groceries (“yes”=1) |  |  |  |
| Difficulty sitting 2 hours (“yes”=1) |  |  |  |
| Difficulty stooping, kneeling or crouching (“yes”=1) | -0.02 | -0.01 |  |
| Difficulty taking medications (“yes”=1) | 0.09 | 0.08 |  |
| Difficulty using map to figure out how to get around strange place (“yes”=1) |  |  |  |
| Difficulty using the toilet, including getting up or down (“yes”=1) | 0.02 | 0.004 |  |
| Difficulty walking 100 yards (“yes”=1) | 0.15 | 0.15 | 0.25 |
| Difficulty walking across a room (“yes”=1) | 0.18 | 0.17 |  |
| Do you find it difficult to follow a conversation (“yes”=1) | -0.12 | -0.11 |  |
| Has/va children (“yes”=1) | -0.28 | -0.28 |  |
| Have you ever fractured your hip? (“yes”=1) | 0.59 | 0.57 |  |
| Have you fallen down in the last two years (for any reason)? (“yes”=1) | -0.10 | -0.09 |  |
| Job status: Professional (“yes”=1) |  | . |  |
| Job status: Skilled manual (“yes”=1) | -0.09 | -0.08 |  |
| Job status: Skilled non-manual (“yes”=1) | 0.01 | 0.001 |  |
| Job status: Unskilled (“yes”=1) | 0.02 | 0.005 |  |
| Hypertension (“yes”=1) | 0.09 | 0.09 |  |
| Limiting longstanding illness (any) (“yes”=1) | 0.13 | 0.13 | 0.05 |
| Lives in urban areas (“yes”=1) | 0.03 | 0.02 |  |
| Living alone (“yes”=1) | 0.01 | 0.004 |  |
| Low level of wealth (“low”=1) | 0.16 | 0.16 | 0.04 |
| Male gender (“male”=1) | 0.50 | 0.50 | 0.03 |
| No qualification (“yes”=1) |  |  |  |
| No vigorous/moderate activity at least once per week (“yes”=1) | 0.09 | 0.09 |  |
| Not in a relationship (“yes”=1) | 0.10 | 0.10 |  |
| Not involved in any organisations (“yes”=1) | 0.12 | 0.12 |  |
| Number of close relationships 1or less (“yes”=1) |  |  |  |
| Number of friends 1 or less (“yes”=1) |  |  |  |
| Number of mobility impairments |  |  |  |
| Owns own house (“yes”=1) | -0.01 | -0.01 |  |
| Poor eyesight (“yes”=1) |  |  |  |
| Poor eyesight for seeing things at a distance (“yes”=1) | 0.00 |  |  |
| Poor eyesight for seeing things up close (“yes”=1) | -0.02 | -0.01 |  |
| Poor hearing (“yes”=1) |  |  |  |
| Poor self-rated health (“yes”=1) | 0.25 | 0.25 | 0.34 |
| Poor self-rated memory (“yes”=1) |  |  |  |
| Shortage of money stops me from doing the things I want to do (“yes”=1) | 0.01 | 0.003 |  |
| Social isolation excluding marriage (“yes”=1) |  |  |  |
| Stroke (“yes”=1) | 0.29 | 0.29 | 0.05 |
| White ethnicity (“yes”=1) | 0.47 | 0.44 |  |

BMI, Body mass index; CASP, Quality of Life Scale (CASP-19)
