# Supplementary material for: Development and validation of prediction model to estimate 10-year risk of all-cause mortality using modern statistical learning methods: a large population-based cohort study and external validation
Source: BMC Med Res Methodol. 2021 Jan 6;21:8. doi: 10.1186/s12874-020-01204-7 (PMC7789636; doi:10.1186/s12874-020-01204-7)
Supplement: Supplementary file 6 — Additional file 6. Estimated optimism. [file 12874_2020_1204_MOESM6_ESM.docx]

**Additional file 6. Estimated optimism**

| Average Optimism | **Model_Best_** | **Model_1-SE_** | **Model_3%_** |
| --- | --- | --- | --- |
| C-index | 0.007614 | 0.007255 | 0.003454 |
| Calibration slope | 0.04963 | 0.047408 | 0.027743 |
|  |  |  |  |
| *At standard threshold* | 50.0% | 50.0% | 50.0% |
| Sensitivity | 0.0016221 | 0.0012424 | 0.0010851 |
| Specificity | 0.0010956 | 0.0010527 | 0.0024796 |
|  |  |  |  |
| *At the best threshold* | 13.2% | 12.6% | 14.9% |
| Sensitivity | 0.0003024 | 0.0004185 | -0.0001093 |
| Specificity | 0.0002441 | 0.0002022 | 0.0001124 |
