# Supplementary material for: Development and validation of prediction model to estimate 10-year risk of all-cause mortality using modern statistical learning methods: a large population-based cohort study and external validation
Source: BMC Med Res Methodol. 2021 Jan 6;21:8. doi: 10.1186/s12874-020-01204-7 (PMC7789636; doi:10.1186/s12874-020-01204-7)
Supplement: Supplementary file 8 — Additional file 8. Optimism-corrected models’ performance in prediction the 10-year risk of all-cause mortality in older adults. [file 12874_2020_1204_MOESM8_ESM.docx]

**Additional file 8. Optimism-corrected models’ performance in prediction the 10-year risk of all-cause mortality in older adults.**

|  | **Model_best_** | **Model_1-SE_** | **Model_3%_** |
| --- | --- | --- | --- |
| Calibration slope | 1.06 | 1.07 | 1.64 |
| C-index | 0.75 | 0.75 | 0.74 |
| Number of predictors selected | 55 | 54 | 13 |
