# Supplementary material for: Development and validation of prediction model to estimate 10-year risk of all-cause mortality using modern statistical learning methods: a large population-based cohort study and external validation
Source: BMC Med Res Methodol. 2021 Jan 6;21:8. doi: 10.1186/s12874-020-01204-7 (PMC7789636; doi:10.1186/s12874-020-01204-7)
Supplement: Supplementary file 10 — Additional file 10. Internally validated though optimism-correction models’ discrimination for prediction the 10-year risk of all-cause mortality in older adults. [file 12874_2020_1204_MOESM10_ESM.docx]

**Additional file 10. Internally validated though optimism-correction models’ discrimination for prediction the 10-year risk of all-cause mortality in older adults.**

|  |  | **Model_best_** | **Model_1-SE_** | **Model_3%_** |
| --- | --- | --- | --- | --- |
| *At standard threshold* | | *50.0%* | *50.0%* | *50.0%* |
|  | Sensitivity | 19.34% | 19.08% | -0.09% |
|  | Specificity | 98.49% | 98.49% | 100% |
|  |  |  |  |  |
| *At the best threshold* | | *13.2%* | *12.6%* | *14.9%* |
|  | Sensitivity | 75.17% | 76.56% | 72.51% |
|  | Specificity | 74.28% | 72.58% | 72.99% |
