# Supplementary material for: Development and validation of prediction model to estimate 10-year risk of all-cause mortality using modern statistical learning methods: a large population-based cohort study and external validation
Source: BMC Med Res Methodol. 2021 Jan 6;21:8. doi: 10.1186/s12874-020-01204-7 (PMC7789636; doi:10.1186/s12874-020-01204-7)

**Additional file 12. The distribution of survival probabilities estimated based on 13 variables included in the model in the development and validation cohorts.**


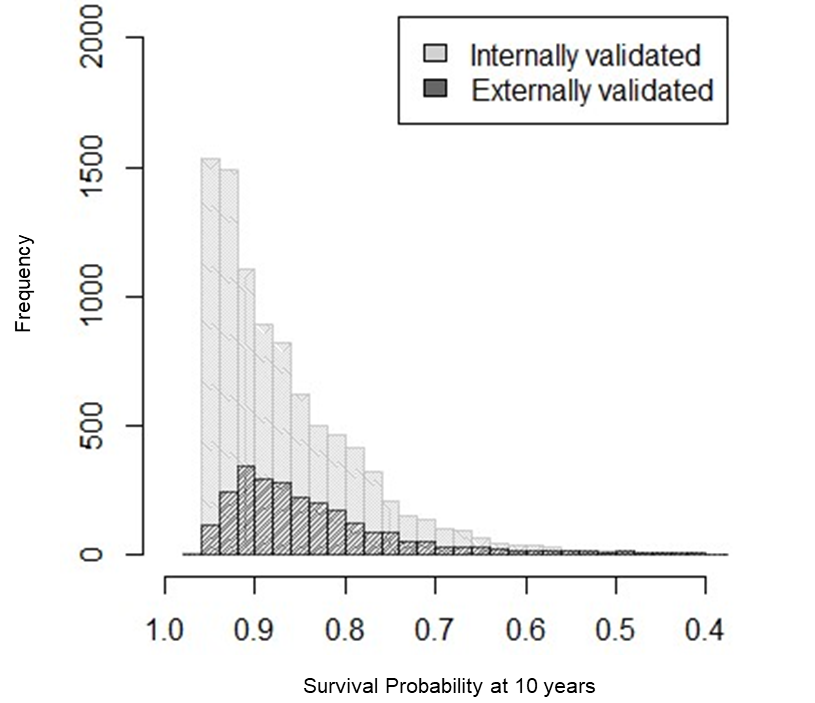

Supplement: Supplementary file 12 — Additional file 12. The distribution of survival probabilities estimated based on 13 variables included in the model in the development and validation cohorts. [file 12874_2020_1204_MOESM12_ESM.docx]
