# Supplementary material for: Development and validation of prediction model to estimate 10-year risk of all-cause mortality using modern statistical learning methods: a large population-based cohort study and external validation
Source: BMC Med Res Methodol. 2021 Jan 6;21:8. doi: 10.1186/s12874-020-01204-7 (PMC7789636; doi:10.1186/s12874-020-01204-7)
Supplement: Supplementary file 13 — Additional file 13. Distributions of the variables included in the final all-cause mortality model in derivation cohort (ELSA) and validation cohort (HRS). [file 12874_2020_1204_MOESM13_ESM.docx]

**Additional file 13. Distributions of the variables included in the final all-cause mortality model in derivation cohort (ELSA) and validation cohort (HRS).**

| **Variables** | **ELSA** | **HRS** |
| --- | --- | --- |
| Age (years), mean (SD) | 61.5 (7.2) | 62.7 (7.2) |
| CASP: I never choose to do things that I have never done before, n (%) | 818 (8.9) | 200 (7.9) |
| Cognition: Memory (score), mean (SD) | 9.9 (3.3) | 10.4 (3.1) |
| Limiting longstanding illness (any), n (%) | 2949 (32.2) | 930 (36.1) |
| Low wealth, n (%) | 3113 (34.0) | 851 (33.1) |
| Male gender, n (%) | 4263 (46.6) | 1109 (43.1) |
| Currently a smoker, n (%) | 1812 (31.1) | 428 (16.8) |
| History of stroke, n (%) | 294 (3.2) | 119 (4.5) |
| Difficulty doing work around house and garden, n (%) | 1172 (12.8) | 404 (15.7) |
| History of cancer, n (%) | 521 (5.7) | 294 (11.4) |
| Difficulty walking 100 yards, n (%) | 887 (9.8) | 270 (10.5) |
| Poor self-rated health, n (%) | 2299 (25.1) | 688 (26.7) |
| Chronic lung disease, n (%) | 533 (5.8) | 277 (10.8) |

ELSA, English Longitudinal Study of Ageing; HRS, Health and Retirement Study; CASP, Quality of Life Scale (CASP-19); SD, standard deviation.
