# Supplementary material for: Development and validation of prediction model to estimate 10-year risk of all-cause mortality using modern statistical learning methods: a large population-based cohort study and external validation
Source: BMC Med Res Methodol. 2021 Jan 6;21:8. doi: 10.1186/s12874-020-01204-7 (PMC7789636; doi:10.1186/s12874-020-01204-7)
Supplement: Supplementary file 14 — Additional file 14. The formula for the final model. [file 12874_2020_1204_MOESM14_ESM.docx]

**Additional file 14. The formula for the final model**

Equation 1. The equation to predict an individual 10-year risk of all-cause mortality in adults aged 50-75 years old using Cox-Lasso model with 2% penalty.

$h\left( \hat{p} \right)$ provides the probability of mortality for each new observation. If the probability is over 14.9%, then the model would classify the observation as died.

$$\log\left( h\left( \hat{p} \right) \right)=h0 + \beta corrected \times liner predictor=+ -0.01159706\times Cognition: Memory (score)+ 0.17867172\times CASP: I never choose to do things that I have never done before \left( “yes”=1 \right) + 0.05317362\times Any limited life conditions \left( “yes”=1 \right)+ 0.04348345\times Low wealth\left( “yes”=1 \right)+ 0.03158487\times Male gender \left( “yes”=1 \right)+ 0.06857408 Currently a smoker \left( “yes”=1 \right)+ 0.05087708\times History of stroke \left( “yes”=1 \right)+ 0.08604836\times Difficulty doing work around house and garden (“yes”=1) + 0.06954402\times Age \left( \mathrm{years} \right)+ 0.11976156\times History of cancer (“yes”=1) + 0.24976016\times Difficulty walking 100 yards (“yes”=1) + 0.33685012\times Poor self-rated health (“yes”=1) + 0.31575449\times Chronic lung disease (“yes”=1)$$
